# Linear and Nonlinear Photon-Induced Cross Bridge/Space Charge Transfer in STC Molecular Crystals

**DOI:** 10.3390/nano12030535

**Published:** 2022-02-04

**Authors:** Chen Lu, Jing Yu, Hao Sheng, Yongjian Jiang, Fengyang Zhao, Jingang Wang

**Affiliations:** 1 College of Science, Liaoning Petrochemical University, Fushun 113001, China; luchen@stu.lnpu.edu.cn (C.L.); yujing3657@sina.com (J.Y.); xiao.jian.happy@163.com (Y.J.); 2 College of Chemistry and Material Science, Liaoning Petrochemical University, Fushun 113001, China; a406280751@163.com

**Keywords:** two-photon absorption, one-photon absorption, cross bridge/space charge transfer, molecular crystals, molecular junctions

## Abstract

In this work, we theoretically studied the optical absorption properties of a layer-stacked cocrystal heterogeneous material Spe-TCNB cocrystal (STC) which is produced by supramolecular self-assembly of organic conjugated monomers SPE and TCNB. The highly ordered aggregate structure in the cocrystal STC will lead to intermolecular interactions such as π∼π, hydrogen bonds and van der Waals forces, resulting in significant charge transfer characteristics and large cross-sectional two-photon absorption characteristics. The physical mechanism of one-photon and two-photon charge transfer of cocrystal molecules is specifically discussed and the interaction between molecules and their role in charge transfer are quantitatively analyzed. We found that the charge transfer between molecular junctions composed of hydrogen bonds is mainly cross-bridge charge transfer, while the charge transfer between molecular junctions caused by accumulation is mainly cross-space charge transfer. This discovery is of great significance to the design of organic photoelectric functional materials.

## 1. Introduction

Two-photon absorption (TPA), a third-order nonlinear optical effect [1,2] has many potential applications in physics [3], chemistry [4,5], and life sciences [6,7], where TPA can be used to excite high-excitation energy systems at long wavelengths for nondestructive photo-catalysis [8,9,10] or to observe physical processes such as two-photon excited fluorescence (TPEF) [11,12,13]. In photo-catalysis, long-wavelength pulsed lasers minimize the damage and thermal effects of the light source on the catalytic substrate, which not only allows for sustainable reactions but also eliminates thermal effects in the studied mechanism. TPEF is a good in vivo bioassay because long-wave light penetrates well into skin tissue. To design systems with good TPA properties, the TPA transition process of polymer systems has been studied in detail by a three-state model [14,15,16] based on the sum-of-states (SOS) method [17]. Some charge-transfer properties in this process are different from those of one-photon absorption (OPA), such as super-exchange and serial charge transfer [18]. However, for the application of TPA, it is essential to discuss the intermolecular TPA charge transfer properties between molecules, even in molecular crystals. This is because the heterogeneous molecules have different front-line orbital energy levels and form molecular junctions co-excited by a light source. In this process, there must be local excitation or charge transfer excitation, which is especially important for the electronic properties of molecular crystals or hetero-molecular co-crystals. For example, for highly stable surface phallocentric compounds as hole transportation layers in perovskites solar cells (PSCs) [19,20]. The intermolecular electron–hole separation properties (charge transfer properties) under illumination are extremely important for device performance. Recently, studies have shown that TCNB can retain the TPA properties of the donor molecule, and that by properly selecting the donor and acceptor molecules, the intermolecular interactions can be controlled, thereby selectively modulating the properties of the co-crystal [21]. Therefore, in this work, we chose a co-crystal composed of Spe and TCNB molecules to discuss the TPA leap characteristics in a heterogeneous molecular constituted co-crystal system. We adopted the crystal structure for the theoretical study to quantify the roles played by molecules at different positions in the super crystalline cell in the charge transfer process, i.e., donor, receiver, or bridge. The ratio of charge transfer across a bridge to space is discussed quantitatively. In addition, the relationship between hetero molecular dipole moments and intermolecular interactions and charge transfer capability in molecular crystals is quantitatively studied.

## 2. Methods

First, we used Gaussian 16 A03 software [22] combined with density functional theory (DFT)-based [23], B3LYP functional [24], and 6-311G (d, p) [25] basis functions to geometrically optimize the crystal structure of individual H elements [26] and extract the monomer molecules for study. Then, based on the optimized structure, the excited state calculations were performed by combining the time-dependent density functional theory (TDDFT) method with the CAM-B3LYP functional [27] and the 6-311G (d, p) basis function. Based on the results of these calculations, the three-state model SOS method calculates the TPA absorption cross-section [15]. The results of this method are in good agreement with the experiments and the quadratic response theory [14,18]. We used a Gaussian function to broaden the OPA and TPA spectrum. The Gaussian function is defined as:

(1) $$G(\omega )=\frac{1}{c\sqrt{2\pi }}e^{-\frac{{\left(\omega -\omega_{i}\right)}^{2}}{2c^{2}}}$$

(2) $$c=\frac{FWHM}{2\sqrt{2ln2}}$$

where ω is the abscissa of the spectrum, $\omega_{i}$ is the transition energy, FWHM is the full width at half maximum.

Finally, the Multiwfn 3.7 [28] program is used to implement the electron–hole pair density, transition density matrix, intermolecular interactions, and cross bridge/space analysis.

## 3. Results and Discussion

### 3.1. Molecular Structure

**Figure 1:**
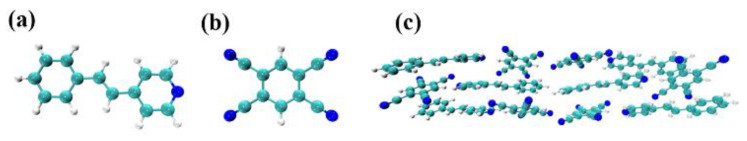
Molecular structure of Spe (**a**), TCNB (**b**), and STC (**c**).

The STC crystal was synthesized by supramolecular self-assembly from monomers 4-styrylpyridine (Spe) and 1,2,4,5-tetracyanobenzene (TCNB). Figure 1c shows a molecular unit of STC. This unit is alternately connected by two Spe and two TCNBs to form a layered structure. These layers are superimposed to form a stacked structure, but this accumulation is staggered due to the differences in molecular length and the arrangement of Spe and TCNB. There are three kinds of intermolecular interactions in the intercepted STC periodic unit (Table 1), among which the interlayer interaction comes from two Spe monomers (3.55 Å) and the π bond formed by Spe and TCNB (3.24 Å). The interaction connecting the stacked layers is CH···N bond (2.04 Å) formed by adjacent Spe and TCNB. Therefore, supramolecular self-assembly shortens the distance between monomer molecules in the STC system. This close bond promotes charge transfer between molecules and enhances the degree of electron delocalization between monomer molecules.

### 3.2. OPA and TPA Spectrum

**Figure 2:**
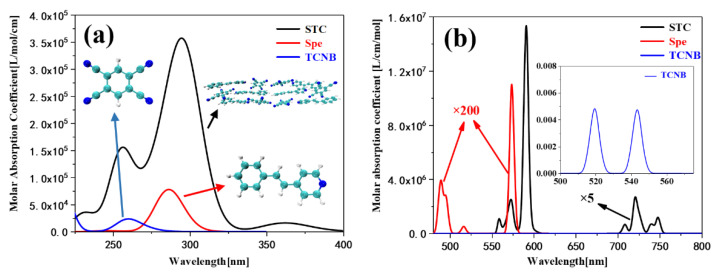
The combined OPA (**a**) and TPA (**b**) Spectrum of STC, Spe, and TCNB (The combined spectrum of some wavelengths in Spe and TCNB have been amplified).

As shown in the black curve of Figure 2a, the OPA Spectrum shows that the STC co-crystal after supramolecular self-assembly has three absorption peaks in the near-ultraviolet region, and there are two strong absorption peaks, of which the absorption peak at 301 nm is mainly composed of two transitions from the ground state to excited states S10 and S14. The combination causes the two excited states, and the absorption peak at 255 nm is caused by S73, see Figure S1a. These two strong absorption peaks are derived from the two monomers. The larger molar absorption coefficient corresponds to the monomer Spe’s absorption peak near 286 nm (red line in Figure 1a and Figure S1c), and the weaker absorption peak corresponds to the monomer TCNB near 259 nm. The absorption peak (blue line in Figure 2a and Figure S1e) is different from the monomer in that the co-crystal STC produces a new weak absorption peak at 360 nm. This absorption peak’s contribution comes from the two transitions from the ground state to excited states S2 and S7, see Figure S1a, which indicates that the two monomers have intermolecular interactions within the STC after the supramolecular self-assembly. It is worth mentioning that STC originates a new absorption band in the near-ultraviolet region at 340 to 400 nm. The absorption peak is of great significance to the analysis of the structure of its molecules.

We found that the TPA spectrum was significantly narrower than the OPA spectrum. There are two main reasons for this, First, the molar absorptivity of OPA and TPA differs by two orders of magnitude. Second, the spectrum of TPA is twice as wide as that of OPA. These cause the absorption peak of the TPA spectrum to appear relatively narrow. The TPA spectrum fitting graph shows that the eutectic STC has a very large molar absorption coefficient (refer to black curve in Figure 1b) and the molar absorption coefficients of Spe and TCNB are small. To clearly analyze the TPA spectrum, the molar absorption coefficient of STC in the range of 700–750 nm is processed by ×5 and the molar absorption coefficient of Spe in the range of 480–600 nm is processed by ×200 (refer to red curve in Figure 1b). The absorption rate is represented by a small graph, where the difference between STC and Spe multiples is 102, and that of TCNB is 109. This also proves that STC can show a large TPA cross-section, while the two monomers do not show a TPA cross-section. The TPA of STC concentrates on two absorption peaks, the strong absorption peak ranges from 550 nm to 600 nm, and its main contribution comes from four transitions from the ground state to excited states S1, S2, S7, and S8. The weak absorption peak ranges from 700 to 750 nm, its primary contributions being three transitions from the ground state to excited states S15, S25, and S34, see Figure S1b. It is worth noting that S2 and S7 are both OPA and TPA excited states. Experiments have proved that the TPA characteristic of STC comes from the intermolecular interaction between Spe and TCNB, and the delocalization of π–*conjugated*. electrons in the entire STC crystal system leads to electronic polarization in the supramolecular structure [28]. TPA Spectroscopy theoretically proves that it is feasible to use supramolecular self-assembly to generate molecular systems with large TPA cross-sections.

### 3.3. Transition Characteristics of OPA

**Figure 3:**
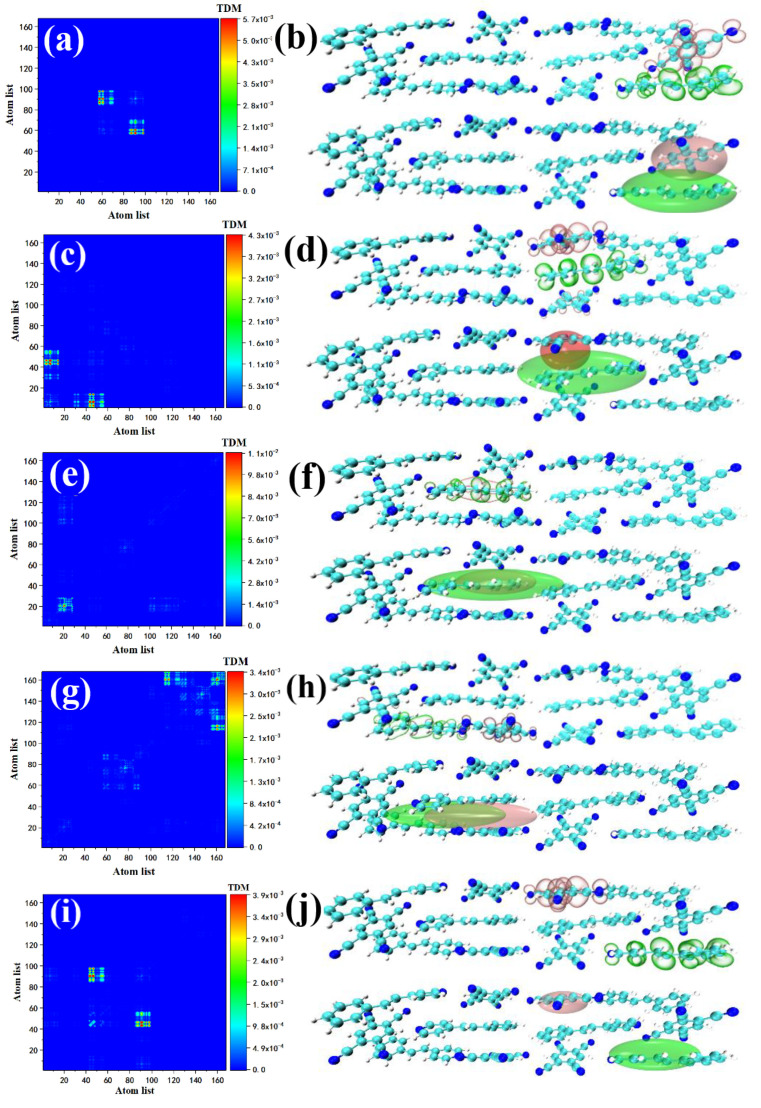
TDM, electron–hole pair density and its *C_hole_* & *C_ele_* of S2 (**a**,**b**), S7 (**c**,**d**), S10 (**e**,**f**), S14 (**g**,**h**), and S34 (**i**,**j**) in STC.

The analysis of intramolecular electron transition behavior is an effective method to study supramolecular polarization in crystal systems [29]. We calculated and plotted the electronic transition density matrix (TDM) of the STC excited state and the electron–hole pair density to investigate the conjugated electron delocalization behavior. TDM is a matrix that contains information about the characteristics of electronic transitions. TDM can reflect the influence of the coupling between different positions of the system on the electronic excitation and can also describe the transition strength. Combined with the electron–hole pair density, the transition characteristics of different excited states can be vividly described. S2 (the second excited state) is the charge transfer excited state, and the electron–hole system is concentrated between Spe and TCNB atoms 83–96 (the atomic numbers are shown in Figure S2, lower right corner, see Figure 3a,b). The transition density matrix of S7 shows that charge transfer transitions occur on the benzene ring of STC and the four cyano groups (atoms 51–64), see Figure 3c. The corresponding electron–hole pair density shows that the electron–hole isosurface is mainly distributed in Spe between the benzene ring and the benzene ring of TCNB, see Figure 3d. This is due to the π–π interaction between the two benzene rings. Because the four cyano groups on TCNB have a strong ability to attract electrons. Therefore, S7 is a charge transfer transition within the crystal molecule. This strong charge transfer comes from the short distance *D*–*π*–*A* interaction after supramolecular self-assembly [30], which leads to electron delocalization in the molecule.

The above analysis of the electronic transition characteristics of the excited state of the large oscillator intensity concentratedly reflects the strong pi–pi* excitation (S2, S7) between the monomer molecules Spe and TCNB, hydrogen bond interaction (S14), weak pi–pi* excitation between two benzene rings on monomer Spe and the strong pi–pi* excitation across spaces (S34) in the STC crystal after supramolecular self-assembly. To quantitatively analyze the electronic excitation characteristics of the characteristic excited states of these interactions, it is necessary to perform wavefunction analysis on these excited states to obtain their transition index, see Table 2. First, we examined the *H index* of S14, which is defined as:

(3) $$H\;index=\left(\left|\sigma_{ele}\right|+\left|\sigma_{hole}\right|\right)/2$$

where *H index* represents the average distribution breadth of holes and electrons. Compared with other excited states, S14 mainly reflects the hydrogen bond interaction characteristics of Spe and TCNB, the electrostatic interaction makes the benzene ring on the Spe lose electrons and appear as holes. The -CN substituent adjacent to Spe on TCNB obtains electrons through electrostatic attraction, and the overall performance is an increase in electron density. Therefore, S14 has the largest spatial distribution breadth (*H index* = 9.937). Because S14 has a small electron–hole isosurface, the charge transfer characteristics are not obvious visually. At this time, the electron and hole distribution trend can be visually displayed by the smoothing of electrons and holes. According to the definition of Tangui Le Bahers [31], the centroid of positive and negative charges ($C_{+}(r)$ &$C_{-}(r)$) is:

(4) $$C_{+}(r)=A_{+}e\left(-\frac{{\left(x-x_{+}\right)}^{2}}{2\sigma_{+x}^{2}}-\frac{{\left(y-y_{+}\right)}^{2}}{2\sigma_{+y}^{2}}-\frac{{\left(z-z_{+}\right)}^{2}}{2\sigma_{+z}^{2}}\right)\;C_{-}(r)=A_{-}e\left(-\frac{{\left(x-x_{-}\right)}^{2}}{2\sigma_{-x}^{2}}-\frac{{\left(y-y_{-}\right)}^{2}}{2\sigma_{-y}^{2}}-\frac{{\left(z-z_{-}\right)}^{2}}{2\sigma_{-z}^{2}}\right)$$

This method can be used to define the centroids of electrons and holes ($C_{hole}$ & $C_{ele}$):

(5) $$C_{ele}(r)=A_{ele}exp\left(-\frac{{\left(x-X_{ele}\right)}^{2}}{2\sigma_{ele,x}^{2}}-\frac{{\left(y-Y_{ele}\right)}^{2}}{2\sigma_{ele,y}^{2}}-\frac{{\left(z-Z_{ele}\right)}^{2}}{2\sigma_{ele,z}^{2}}\right)\;C_{hole}(r)=A_{hole}exp\left(-\frac{{\left(x-X_{hole}\right)}^{2}}{2\sigma_{hole,x}^{2}}-\frac{{\left(y-Y_{hole}\right)}^{2}}{2\sigma_{hole,y}^{2}}-\frac{{\left(z-Z_{hole}\right)}^{2}}{2\sigma_{hole,z}^{2}}\right)$$

where *A* is the normalization coefficient, *x*, *y*, *z* are the three Cartesian components of the coordinate vector *r*, and *σ* is the root mean square deviation (RMSD) of the distribution of holes or electrons in the directions of these three components. It reflects the breadth of distribution, and the isosurface value is set to 0.0005, which will be displayed as an ellipse after graphical, see the lower part of Figure 3b,d,f,h,j. It can be seen that the positive and negative values of *C_hole_* and *C_ele_* of S14 have partially overlapped. The *D index* represents the distance of charge transfer and is defined as:

(6) $$\begin{array}{l} D_{x}=\left|X_{ele}-X_{hole}\right|\;D_{y}=\left|Y_{ele}-Y_{hole}\right|\;D_{z}=\left|Z_{ele}-Z_{hole}\right|\\ D_{index}=\sqrt{{\left(D_{x}\right)}^{2}+{\left(D_{y}\right)}^{2}+{\left(D_{z}\right)}^{2}} \end{array}$$

where *X_hole_* refers to the X coordinate of the hole’s centroids, which is obtained by multiplying the $\rho_{hole}$ function by the *x* coordinate variable and integrating in the whole space. The main part of the distribution becomes farther with the increase in *D index*. The *D index* of S34 reaches 10.150 Å. As shown in Figure 3j, the electron–hole pair density shows that the electron–hole isosurface is mainly distributed between the two Spes in the right half of the STC. At this time, it can be clearly seen that *C_hole_* and *C_ele_* have the characteristics of cross-space distribution. This cross-space charge transfer distribution greatly weakens the attraction between electrons and holes, so S34 has the smallest coulomb attraction energy (1.566 eV). In fact, this charge transfer can also be characterized by the degree of hole–electron separation (*t index*). The *t index* is defined as:

(7) $$t\;index=D\;index-H_{CT}$$

where $H_{CT}=\left|H\cdot u_{CT}\right|$ represents the average extent of holes and electrons in the CT direction. It can be seen that the t index of S14 reaches 6.503 Å, which is much larger than other excited states, indicating that the electron holes are highly separated. Contrary to the *t index*, the electron excitation characteristics can also be investigated by the degree of electron–hole overlap (*S_m_*). First, get the *S_m_*(*r*) function by taking the minimum of holes-electrons.

(8) $$S_{m}(r)=\min[\rho^{hole}(r),\rho^{ele}(r)]$$

Secondly, integrate the whole space of *S_m_*(*r*) to get the *S_m_ index*:

(9) $$S_{m}\;index=\int S_{m}(r)dr=\int \min[\rho^{hole}(r),\rho^{ele}(r)]dr$$

The value range of *S_m_ index* is [0, 1]. The larger the value, the higher the degree of electron–hole overlap. It can be seen that the *S_m_ index* of S2 and S7 are only 0.05116 and 0.06812, which further verifies that the electron–hole isosurfaces in the electron hole density diagram having almost no overlap. This shows that S2 and S7 are excited states of charge transfer caused by the strong pi–pi* excitation between the benzene ring of Spe and TCNB. The electronic transitions of these two excited states and S34 both reflect the characteristics of charge transfer. The difference is that is that the *t index* of S2 and S7 is much smaller than that of S34. From the *C_hole_* and *C_ele_* distributions of the two excited states, it can be clearly seen that the centroid distance of the hole-electron in S34 is much larger than that of S2 and S7, see Figure 3b,d,j. The *S_m_ index* of S10 reaches 0.56443, indicating that half of the hole-electron are overlapped, and the negative values of *C_hole_* and *C_ele_* completely wrap the positive values. At the same time, the *t index* is negative, which further shows that the hole–electron pairs are not completely separated. This is in good agreement with the feature that S10 is a local excitation between the two benzene rings inside the Spe monomer, see Figure 3f. The above transition index shows quantitatively the excitation characteristics of electronic transition from many aspects. To investigate the delocalization characteristics of the hole –electron pairs, the hole delocalization index (*HDI*) and the electron delocalization index (*EDI*) are defined:

(10) $$\begin{array}{l} HDI=100\times \sqrt{\int {\left[\rho^{hole}(r)\right]}^{2}dr}\\ EDI=100\times \sqrt{\int {\left[\rho^{ele}(r)\right]}^{2}dr} \end{array}$$

the smaller the values of *EDI* and *HDI*, the higher the degree of hole/electron delocalization, which means the greater uniformity of the distribution. The *HDI* and *EDI* indexes of the charge transfer excited states S2, S7, and S34 are much larger than those of the LE excited state indicating that the degree of delocalization of these electron holes is relatively small, and they are confined to the acceptor and donor, respectively. It reflects the characteristics of supramolecular self-assembly leading to supramolecular polarization, which makes the strong cross-space charge transfer between the organic conjugated monomers in the STC.

Based on the transition density matrix and the electron–hole pair density analysis of S2 and S7, it can be said that this strong *D*–*π*–*A* interaction is the main factor leading to the increase in the degree of electron delocalization in the crystal system. S10 is the local excitation in the crystal molecule. TDM and the electron–hole pair density show that the electron–hole isosurfaces overlap and are evenly distributed on the entire Spe, see Figure 3e,f. This may be due to the interaction between the benzene ring and the pyridine on the Spe. The effect leads to weak electronic transitions. S14 is also an excited state for charge transfer. TDM shows that a small part of the electron transitions occurs inside the molecule, see Figure 3g. The electron–hole pair density indicates that there are still tiny traces on the Spe where the hole density is concentrated and the methyl chain connecting the pyridine and benzene rings. The electron density and this excitation intensity are much smaller than S7, see Figure 3h. This is because this charge transfer is caused by hydrogen bonds (CH···N). S34 ( the 34th excited state) belongs to local excitation, and the distribution of the electron–hole isosurface on the STC is tiny, indicating that the degree of electronic transition is small, see Figure 3i. The TDM and the electron–hole pair density of Spe in S1 and S4 show that the electronic transition of Spe under the near-ultraviolet light excitation belongs to local excitation, see Figure S3a–d. The TDM and the electron–hole pair density of TCNB in S1 and S2 show that the electronic transition of TCNB under near-ultraviolet light excitation belongs to local excitation, see Figure S3e–g,h. As mentioned above, in the TDM and the electron–hole pair density analysis of the one-photon transition of crystal STC, we concluded that the two monomers only exhibit local excitation under the light excitation in the 340–400 nm wavelength range. However, the new crystal obtained after the self-assembly of the two monomers demonstrates strong charge transfer. The optical excitation characteristics of STC are significantly enhanced compared with the two monomers. Electronic transition is the main contributor to the strong charge transfer-excited states due to the strong interaction between Spe and TCNB molecules.

### 3.4. Transition Characteristics of TPA

To deeply analyze the nonlinear optical properties of the crystal in which TCNB obtains a strong charge transfer TPA excited state by mixing and stacking, we further investigated the absorption characteristics of STC and found that STC has a large TPA cross-section. In daily life, organic conjugated chromophores based on TPA cross-sections are widely used in three-dimensional fluorescence imaging, optical data storage, optical limiting, and photolithography micromachining. Therefore, it is of great significance to study the causes of large TPA cross-sections in molecular systems.

**Figure 4:**
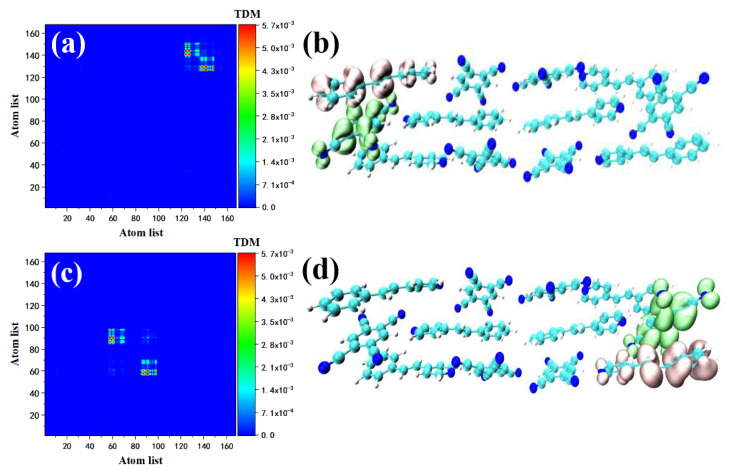
TDM and electron–hole pair density of STC in two-photon transition at 748 nm (**a**,**b**) and 740 nm (**c**,**d**).

We use TDM and the electron–hole pair density to analyze the intermediate state of the two-photon transition’s excitation characteristics. This is because TDM is a useful tool for analyzing the charge transfer between molecules. Simultaneously, compared with the orbital analysis, the contribution of the electron–hole pairs to TPA excited states can better reflect the charge transfer from the ground state to the intermediate state and then to the final state, thus more clearly showing the local and overall excitation characteristics. Both S1 and S2 are excited by strong charge transfer, see Figure 4. Consistent with previous studies, S2 is both an OPA excited state and a TPA excited state. The position of the charge transfer is shifted from the upper left to the lower right corner. This is due to the quantum repulsion effect. The energy levels are split to produce different energy levels. The electronic transition still comes from the strong *D*–*π*–*A* interaction, while the difference in TDM is due to the atomic number. S1 and S2 are essentially the same charge transfer excitation mode. The TPA peaks of STC ranging from 700 to 730 nm include two transitions from the ground state to excited states S7 and S8. The contribution of the two-photon transition in the 715–735 nm range is provided by multiple excited states.

**Figure 5:**
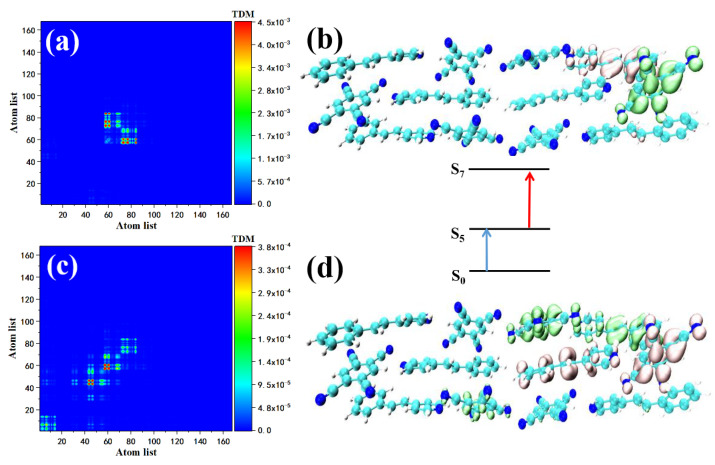
TDM and electron–hole pair density with the two-photon transition of STC at 720 nm, where the first transition is S0–S5 (**a**,**b**) and the second transition is S5–S7 (**c**,**d**).

**Figure 6:**
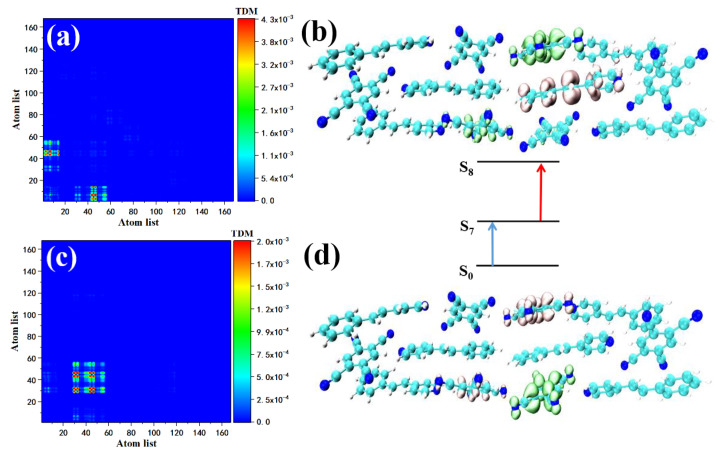
TDM and electron–hole pair density of the two-photon transition of STC at 709 nm, the first transition if S0–S7 (**a**,**b**) and the second transition is S7–S8 (**c**,**d**).

We also investigated the maximum oscillator strength in S7. The first transition of S7, S0→S5, can be represented by Figure 5a,b. According to the transition density matrix, this transition may belong to charge transfer excitation. Combined with the map, the green sound isosurface represents the place where the electron density decreases, expressed by holes; the red isosurface represents the place where the electron density increases, expressed by electrons. The distribution of the isosurface proves that the Spe at the upper right of the STC intermolecular charge transfer excitation occurred between Spe and TCNB (Spe-TCNB unit). The whole TCNB was covered with holes due to the loss of electrons, and the increased electrons were distributed on the benzene ring of Spe. The transition of S5→S7 can be shown in Figure 5c,d. The transition density shows that the range of charge transfer is further expanded, and electron–hole diffuse to the left from one Spe-TCNB unit to another Spe-TCNB unit. Compared with S7, the two-photon excitation characteristics in the 700–710 nm range are contributed by S8 alone. The transition from the ground state to S7 has been explained in the one-photon absorption section, see Figure 6a,b. We mainly analyzed the transition of S7–S8. The density matrix shows that this is a charge transfer excitation, see Figure 6c, and the electron–hole distribution proves that STC has a good absorption of light at this frequency, see Figure 6d.

**Figure 7:**
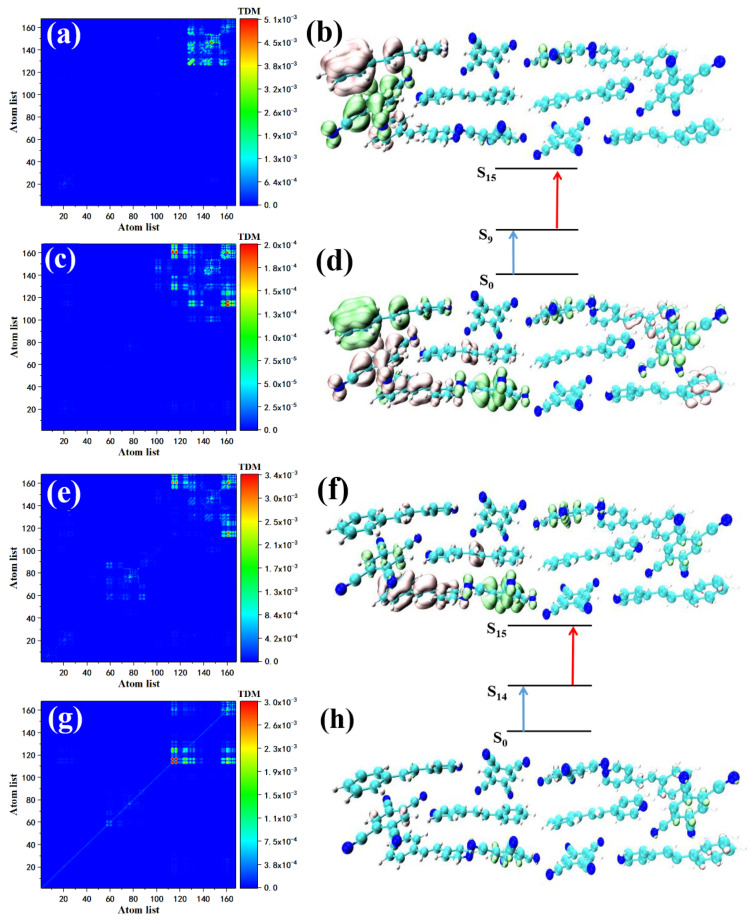
The two-photon transition TDM and electron–hole pair density of STC at 591 nm, where S9 is the intermediate state, the first transition is S0–S9 (**a**,**b**), the second step is S9–S15 (**c**,**d**); S14 is the two-photon transition of the intermediate state, the first step is S0–S14 (**e**,**f**), the second step is S14–S15 (**g**,**h**).

**Figure 8:**
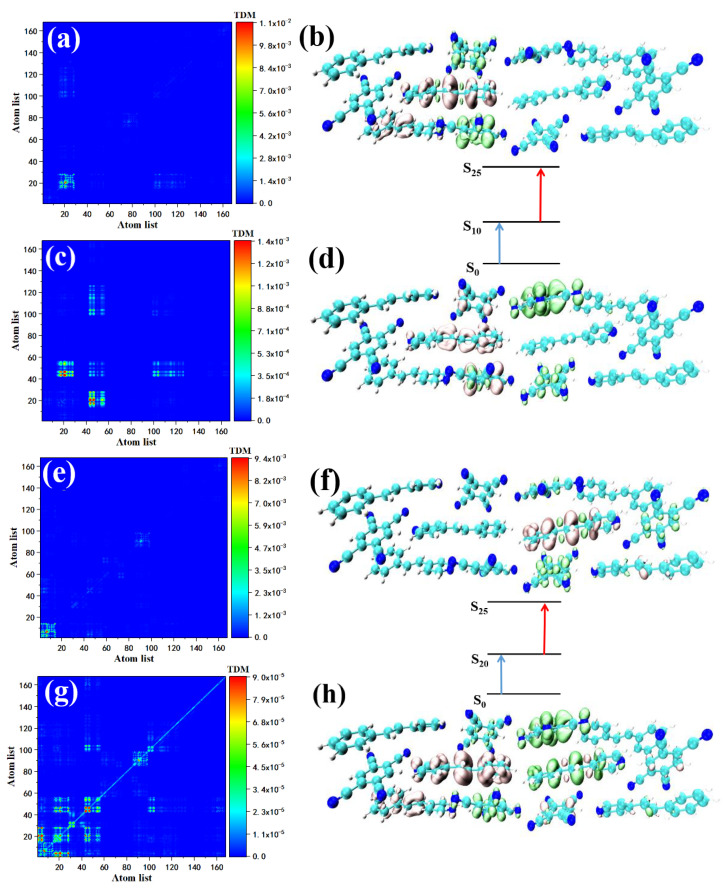
The two-photon transition TDM and electron–hole pair density of STC at 573 nm, where S10 is the intermediate state, the first transition is S0–S10 (**a**,**b**), the second step is S10–S25 (**c**,**d**); S20 is the two-photon transition of the intermediate state, the first step is S0–S20 (**e**,**f**), the second step is S20–S25 (**g**,**h**).

**Figure 9:**
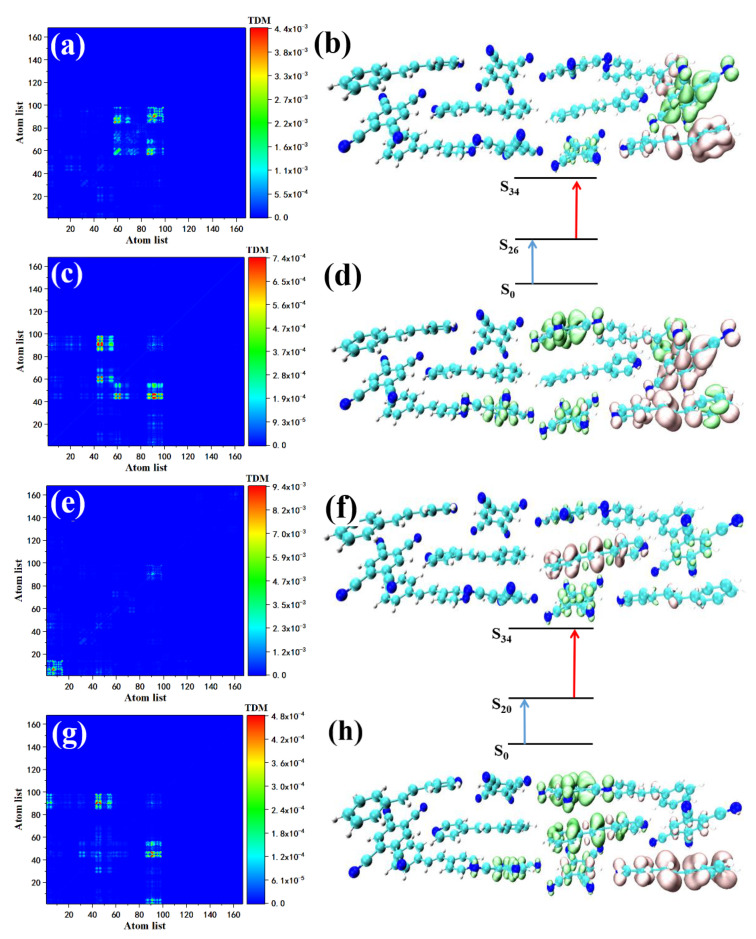
The two-photon transition TDM and electron–hole pair density of STC at 557 nm, when S26 is the intermediate state, the first transition is S0–S26 (**a**,**b**), the second step is S26–S34 (**c**,**d**); when S26 is the intermediate state, the first step is S0–S20 (**e**,**f**), the second step is S20–S34 (**g**,**h**).

STC’s strong absorption peak is between 550–600 nm, including one main peak (S15) and two secondary peaks (contributed by S25 and S34). The two-photon transition of S15 consists of two channels, that is, two intermediate states. First, we analyzed the two-photon transition with S9 as the intermediate state. The first transition is the transition from the ground state to S9. The TDM shows that the transition may have a local effect. Local excitation characteristics combined with the isosurface of the electron–hole pair density, the Spe at the upper left of the STC section shows the electron region, see Figure 7a, and the corresponding TCNB shows holes region, see Figure 7b. The S9→S15 constitutes the second transition of S15. From the perspective of TDM, this is a strong charge transfer excitation, see Figure 7c. The electron–hole pair density shows that the isosurface of the electron region and the distribution of hole isosurface is in the first diffusion to Spe and TCNB at the bottom right, based on step transition, see Figure 7d. This is because the first step of local excitation is the process of charge accumulation, and this charge accumulation is the condition for the second step of charge transfer. S14 is another intermediate state of S15, in which the transition from the ground state to S14 (refer to the excitation properties of S14) is a strong charge transfer excitation, see Figure 7e,f. TDM shows that the local excitation characteristics of the second transition are significant. This kind of charge transfer excitation becomes local excitation because the charge transfer increases the state density of the orbital occupied by the valence band, making the second step of electronic transition produce local excitation. Using the same method to analyze the two-photon transition of the two sub-peaks, the intermediate state of the first channel in S25 is S10, and the first transition S0→S10 is local excitation, see Figure 8a. The electron region and the hole are located in STC on the two Spes and the TCNB at the bottom right of the system, see Figure 8b. There is also a small number of holes on the Spe in the middle, see Figure 8b. The second transition S10→S25 is a strong charge transfer excitation, see Figure 8c. The middle part of the STC unit has significant charge transfer characteristics, see Figure 8d. The intermediate state of the second channel is S20. The first transition is the local excitation, see Figure 8e. There are a small number of holes on the Spe in the middle, see Figure 8b. The second transition S10→S25 is a strong charge transfer excitation, see Figure 8c. The middle part of the STC unit has significant charge transfer characteristics, see Figure 8d. The intermediate state of the second channel is S20. The first transition is the local excitation, see Figure 8e. The overlap of the Spe electron region and the hole located in the middle of the STC periodic unit to the right also proves this, see Figure 8f. The second step transitions into a strong charge transfer excitation, see Figure 8g, and the electronic region diffuses to the monomer Spe on the right in the middle of the STC. At the same time, electron delocalization occurred between the Spe and TCNB below the monomer, see Figure 8h. The two TPA transition channels of S25 are local excitation, and strong charge transfer excitation is composed of two transition processes. The contribution of the two-photon transition of the second peak comes from S34. The excitation characteristic of S34 is composed of two channels. The intermediate state of the first channel is S26. The transition of S0→S26 belongs to local excitation, see Figure 9a,b. The second step’s transition characteristic is dominated by charge transfer excitation, see Figure 9c,d. Another intermediate state of S34 is S20. Combining Figure 9e,f, we can know that the first transition belongs to local excitation. The two-step transition belongs to the charge transfer excitation, see Figure 9g,h, so it can be concluded that both S28 and S34 have two channels, and the excitation characteristics of the two-step transition of each channel are local excitation to strong charge transfer excitation. This is because the two-step transition in the two-photon transition will change the weaker absorption coefficient of charge transfer excitation so that local excitation can enhance the charge transfer excitation, and charge transfer may become the main absorption peak.

### 3.5. Cross Bridge/Space Charge Transfer Analysis

There are two forms of electronic transition in the system when local excitation or charge transfer excitation occurs in the molecule: cross space charge transfer (CSCT) and cross bridge charge transfer (CBCT). In analyzing the charge transfer in the excited states of STC molecules, the mechanism needs to be considered from two perspectives. First, when the molecule reaches the excited state, the charge transfer’s contribution is mainly from the non-relaxed part, corresponding to the hole and electron distribution. The visualized result is the electron–hole pair density. For this concept of charge transfer, the electronic excitation can be calculated by the interfragment charge transfer (IFCT) method. The amount of electron transfer between any fragments in the process, the direct charge transfer among which can be regarded as cross space, and the charge transfer through *π*–*A*, *A*–*π* can be regarded as cross bridge. On the other hand, if there is a transition forbidden during the electron transition, there is no spatial overlap between holes and electrons, even if the degree of charge transfer is large, it will not be observed. This part of the charge transfer does not contribute to light absorption and emission. It is necessary to calculate whether the some of the electrons contributing to the absorption and emission of light in the charge transfer transition are distributed in the space between *D*–*A* or on the bridge between *D*–*A*, determined by the transition density and the hole. The distribution of electron overlap can be used to examine the ratio of CBCT and CSCT.

**Figure 10:**
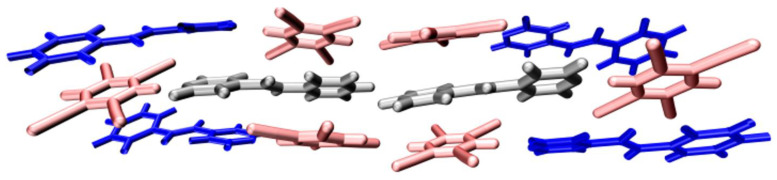
Bridge fragment (black), donor fragment (blue), and acceptor fragment (red) of STC in CB/CSCT.

CB/CSCT analysis needs to define bridging fragments, donor fragments, and acceptor fragments, respectively. When calculating the CBCT and CSCT of the STC cycle unit, the two Spe monomers in the center are used as the bridge, marked with black, and the Spe monomer connecting the bridge is the donor fragment, drawn in blue, and the TCNB monomer is the acceptor fragment, marked in red, see Figure 10. Table 3 shows the CB/CS power calculated by STC using IFCT, transition density, and hole-electron overlap distribution and their respective proportions in the charge transfer volume. From the point of view of the charge transfer value, the total amount of IFCT (0.96738) is significantly more than that due to transition density (0.29008) and hole-electron overlap (0.16522). This is because IFCT analysis is based on the contribution of fragments to holes and electrons. The calculation is the redistributed electrons between the donor fragment and the acceptor fragments in the STC molecule due to light excitation, where transition density and hole-electron overlap does not include the part where holes and electrons do not overlap in space. The proportion of CSCT is much larger than that of CBCT, indicating that the contribution of the charge transfer between *D*–*π*–*A* mainly comes from CSCT, which confirms that the charge transfer between STC molecules is due to the closer Spe monomer and the stronger molecule between TCNB interactions promote electron delocalization.

### 3.6. Analysis of the Molecular Dipole Moment

**Figure 11:**
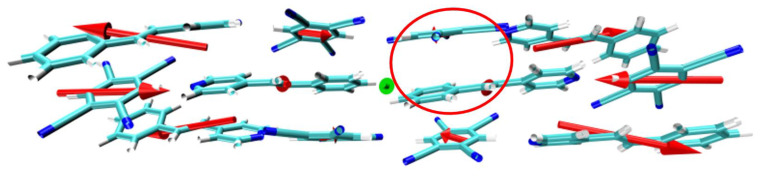
The overall dipole moment of STC (green arrow) and each monomer’s fragment dipole moment (red arrow).

The molecular dipole moment plays an important role in analyzing the polarity of local molecules. In the two-photon transition, the first transition is more likely to occur than the second transition. Table 4 shows the main two-photon transition excited states of STC and their transition dipole moments. The dipole moment of the first transition is greater than the second transition. This is because an enormous dipole moment difference can promote charge transfer between molecules. In the spectral analysis of Section 3.2, the OPA spectrum showed that in the range of 340–380 nm, STC produced a new absorption peak relative to the two monomers. S7 mainly contributed to the absorption peak. TDM and electron–hole pair density analysis shows that S7 is a strong charge transfer excited state. The analysis of TDM and electron–hole pair density of two-photon transitions shows that S7 has strong charge transfer performance. To further study the source of the strong charge transfer characteristics of the eutectic STC in S7, we used the visualization method to plot the overall dipole moment of the STC system, see the green arrow in Figure 11, and the permanent dipole moment of each monomer, see the red arrow in Figure 11. The red circle is the strong charge transfer site of S7. Taking the geometric center of the eutectic STC as the origin, it can be observed that the overall dipole moment of the STC is greater than zero, and the direction is from the outside to the inside. The fragment dipole moment of the single Spe in the red coil points from the outside to the rear left through the paper, while the TCNB’s direction of the dipole moment is from the inside to the right front. Remarkably, the centrally symmetric monomers have opposite and larger dipole moments. This strong intermolecular dipole induction induces the opposite dipole moments of the molecules inside the crystal STC, that is, the permanent couple of S7 in the red coil polar moment, so that the D−π−A system obtains a significant dipole moment difference. It can be known from the TPA section formula, [15], that the cross-section of TPA increases with the increase in dipole moment, which also explains the strong charge transfer characteristics of S7.

## 4. Conclusions

In this work, we used first principles to calculate and analyze the optical absorption characteristics of a molecular crystal material STC with a stacked aggregate structure. We discussed the relationship between one-photon and two-photon absorption properties, intermolecular dipole moments and non-covalent interactions, and charge transfer mechanisms. Supramolecular self-assembly effectively shortens the distance between organic conjugated monomers, so that abundant intermolecular interactions such as *π*–*π* and hydrogen bonds are generated in the STC molecular crystal, which promotes the charge transfer between monomer molecules, thereby enhancing the degree of electron delocalization between monomer molecules in the crystal. The charge transfer between molecules in molecular crystals is mainly realized by CSCT, where *D*–*π*–*A* is the source of dipole induction of monomer molecules in STC. This dipole induction produces an excellent dipole moment difference in the middle part of the SPE-TCNB unit, which results in a larger TPA cross-section in the STC, which further promotes the charge transfer between molecules. The composition of different excited states in cross-space charge transfer and cross-bridge charge transfer of molecular crystals is different, which will provide new ideas for the purposeful design of large TPA cross-section chromophores and electron transport materials.

## Figures and Tables

**Table 1:** Three kinds of intermolecular interactions in the STC crystal, molecular distance, and hydrogen bond length.

	Intermolecular Interaction	Molecular Distance[Å]	Hydrogen Bond Length[Å]
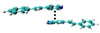	Spe···Spe	3.55	--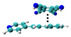	Spe···TCNB	3.24	--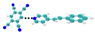	C–H···N	--	2.04

**Table 2:** The transition index of the main OPA excited states.

Excited States	H(Å)	D (Å)	t (Å)	Sm	E (eV)	HDI	EDI
S0→S2	2.971	3.204	0.260	0.05116	3.431	7.36	8.47
S0→S7	3.883	2.545	0.260	0.06812	3.393	6.85	6.40
S0→S10	6.881	3.846	−3.535	0.56443	2.701	5.47	4.23
S0→S14	9.937	3.846	−5.391	0.30617	2.000	4.86	3.73
S0→S34	4.732	10.150	6.503	0.09034	1.566	6.52	7.06

**Table 3:** Analysis methods of CB/CSCT, the amount of charge transfer of CB/CSCT and their respective proportions of total charge transfer.

Analytical Method	IFCT	Transition Density	Hole-Electron Overlap
Through Space CT	0.96738	0.29008	0.16522
	99.98%	98.50%	99.27%
Through Bond CT	0.00016	0.00440	0.00121
	0.02%	1.49%	0.72%
Tot Charge Trans	0.96754	0.29448	0.16643

**Table 4:** The main two-photon transition excited states of STC and their transition dipole moments.

TPA States	Process	Integral Value
S2	$\langle \phi_{S_{0}}\left|\mu \right|\phi_{S_{1}}\rangle \to \langle \phi_{S_{1}}\left|\mu \right|\phi_{S_{2}}\rangle$	0.23→0.00022
S7	$\langle \phi_{S_{0}}\left|\mu \right|\phi_{S_{5}}\rangle \to \langle \phi_{S_{5}}\left|\mu \right|\phi_{S_{7}}\rangle$	0.21→3.47
S8	$\langle \phi_{S_{0}}\left|\mu \right|\phi_{S_{7}}\rangle \to \langle \phi_{S_{7}}\left|\mu \right|\phi_{S_{8}}\rangle$	1.08→14.25
S15	$\langle \phi_{S_{0}}\left|\mu \right|\phi_{S_{14}}\rangle \to \langle \phi_{S_{14}}\left|\mu \right|\phi_{S_{15}}\rangle$	8.53→63.11
	$\langle \phi_{S_{0}}\left|\mu \right|\phi_{S_{9}}\rangle \to \langle \phi_{S_{9}}\left|\mu \right|\phi_{S_{15}}\rangle$	1.31→0.63
S25	$\langle \phi_{S_{0}}\left|\mu \right|\phi_{S_{14}}\rangle \to \langle \phi_{S_{14}}\left|\mu \right|\phi_{S_{25}}\rangle$	16.04→3.81
	$\langle \phi_{S_{0}}\left|\mu \right|\phi_{S_{20}}\rangle \to \langle \phi_{S_{20}}\left|\mu \right|\phi_{S_{25}}\rangle$	4.79→2.56
S34	$\langle \phi_{S_{0}}\left|\mu \right|\phi_{S_{26}}\rangle \to \langle \phi_{S_{26}}\left|\mu \right|\phi_{S_{34}}\rangle$	4.91→3.29
	$\langle \phi_{S_{0}}\left|\mu \right|\phi_{S_{20}}\rangle \to \langle \phi_{S_{20}}\left|\mu \right|\phi_{S_{34}}\rangle$	4.79→0.50

## Data Availability

Data can be available upon request from the authors.

## Supplementary Materials

The following supporting information can be downloaded at: https://www.mdpi.com/article/10.3390/nano12030535/s1, Figure S1: OPA and TPA spectra of STC (a,b), monomer Spe (c,d) and monomer TCNB (e,f); Figure S2: Atomic numbers in STC co-crystals; Figure S3: The monomer Spe’s TDM and electron-hole pairs density of S_1_ (a,b) and S_4_ (c,d); the monomer TCNB’s TDM and electron-hole pairs density of S_1_ (e,f) and S_2_ (g,h).
