# Supplementary material for: Linear and Nonlinear Photon-Induced Cross Bridge/Space Charge Transfer in STC Molecular Crystals
Source: Nanomaterials (Basel). 2022 Feb 4;12(3):535. doi: 10.3390/nano12030535 (PMC8840255; doi:10.3390/nano12030535)
Supplement: Supplementary file 1 [file nanomaterials-12-00535-s001.zip › nanomaterials-1569773-supplementary.pdf]

nanomaterials

Article

Linear and Nonlinear Photon-Induced Cross Bridge/Space Charge Transfer in STC Molecular Crystals

Chen Lu [1], Jing Yu [1], Hao Sheng [1,*], Yongjian Jiang [1], Fengyang Zhao [2] and Jingang Wang [1,*]

1 College of Science, Liaoning Petrochemical University, Fushun 113001, China; luchen@stu.lnpu.edu.cn (C.L.); yujing3657@sina.com (J.Y.); xiao.jian.happy@163.com (Y.J.)
2 College of Chemistry and Material Science, Liaoning Petrochemical University, Fushun 113001, China; a406280751@163.com
* Correspondence: shenghao@lnpu.edu.cn (H.S.); jingang_wang@lnpu.edu.cn (J.W.)

Citation: Lu, C.; Yu, J.; Sheng, H.; Jiang, Y.; Zhao, F.; Wang, J. Linear and Nonlinear Photon-Induced Cross Bridge/Space Charge Transfer in STC Molecular Crystals. *Nanomaterials* **2022**, *12*, 535. https://doi.org/10.3390/nano12030535

Academic Editor: Rodolphe Antoine

Received: 9 January 2022
Accepted: 2 February 2022
Published: 4 February 2022

Publisher's Note: MDPI stays neutral with regard to jurisdictional claims in published maps and institutional affiliations.

Copyright: © 2022 by the authors. Licensee MDPI, Basel, Switzerland. This article is an open access article distributed under the terms and conditions of the Creative Commons Attribution (CC BY) license (https://creativecommons.org/licenses/by/4.0/).

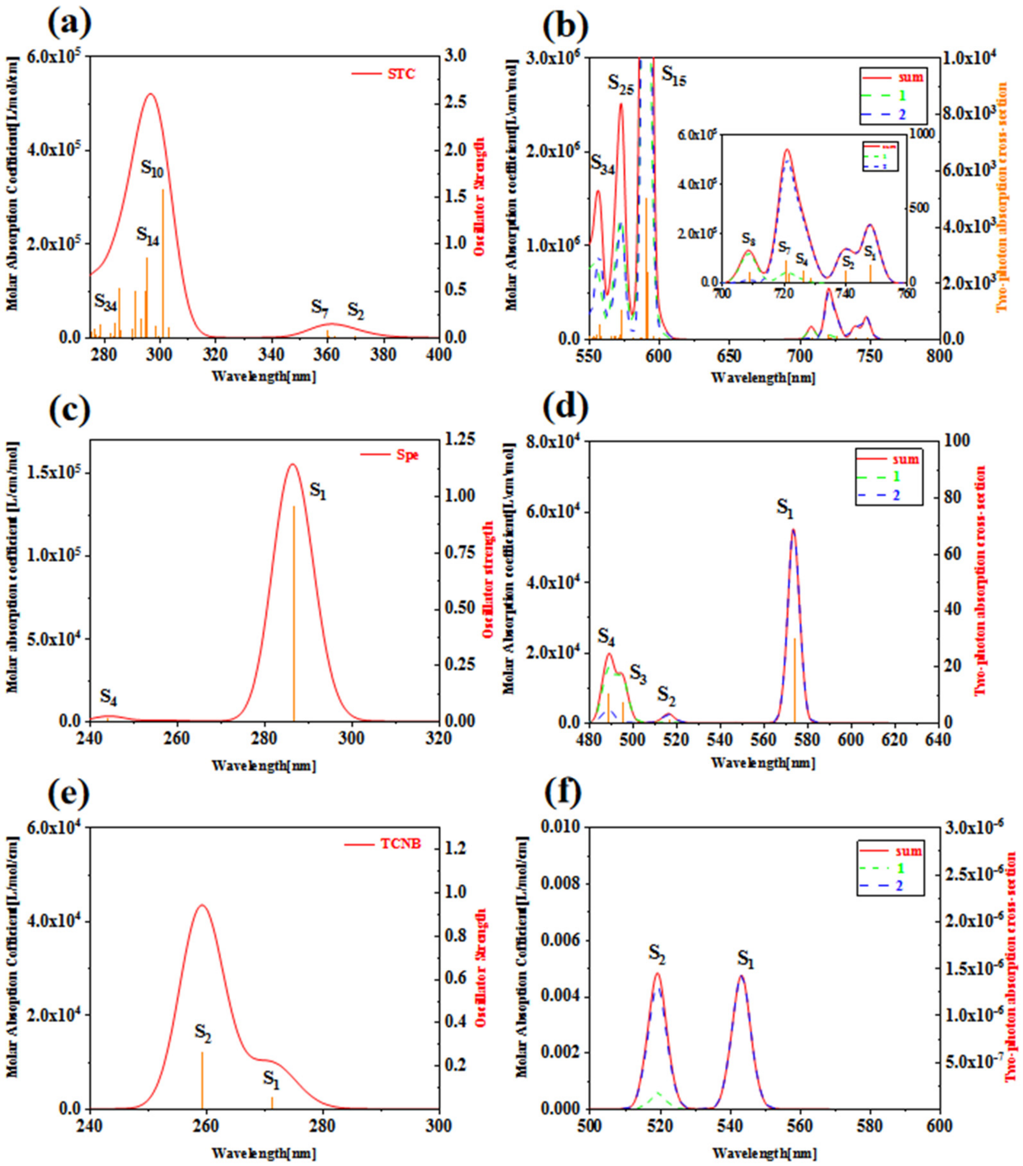

Figure S1. OPA and TPA spectra of STC (a, b), monomer Spe (c, d) and monomer TCNB (e, f).

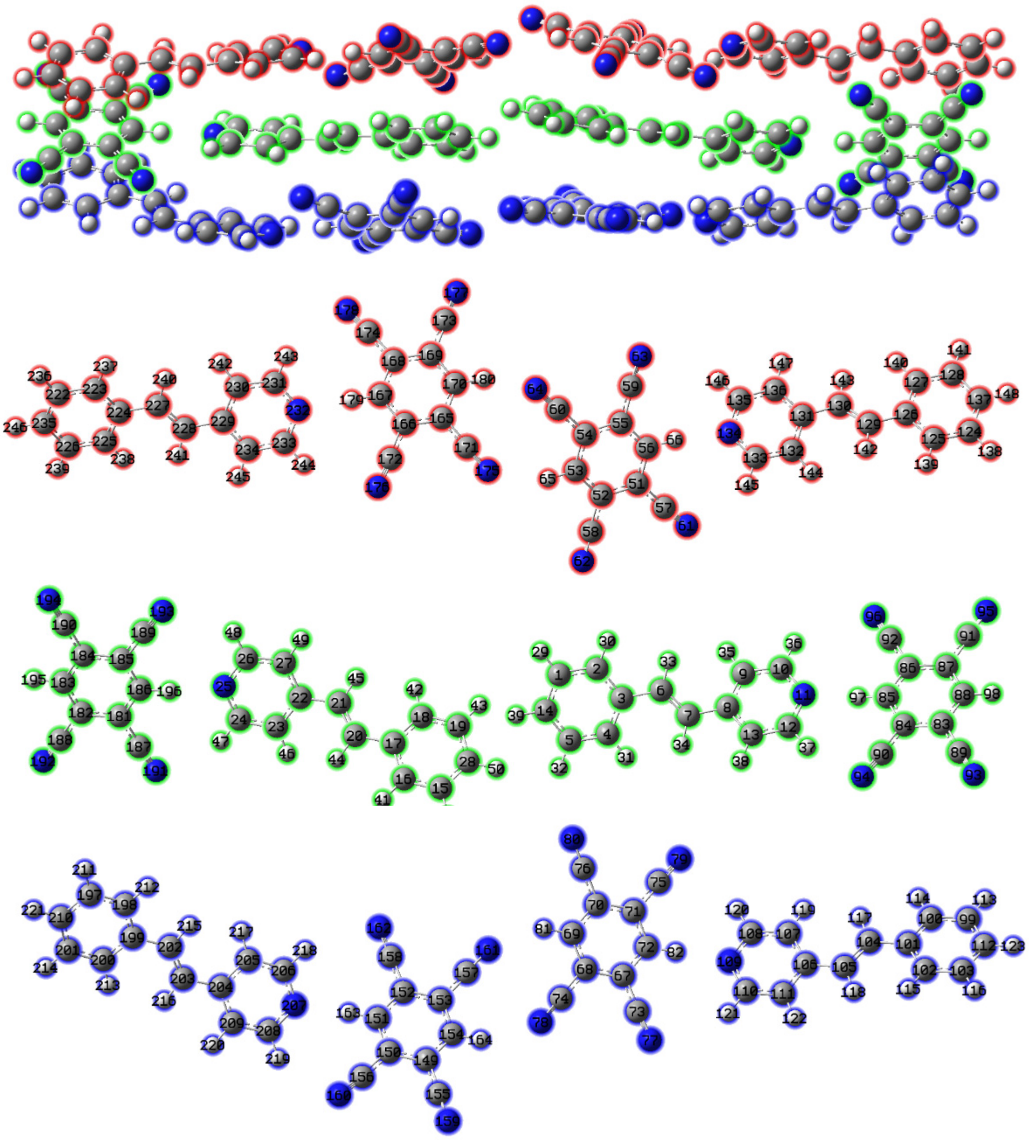

Figure S2. Atomic numbers in STC co-crystals.

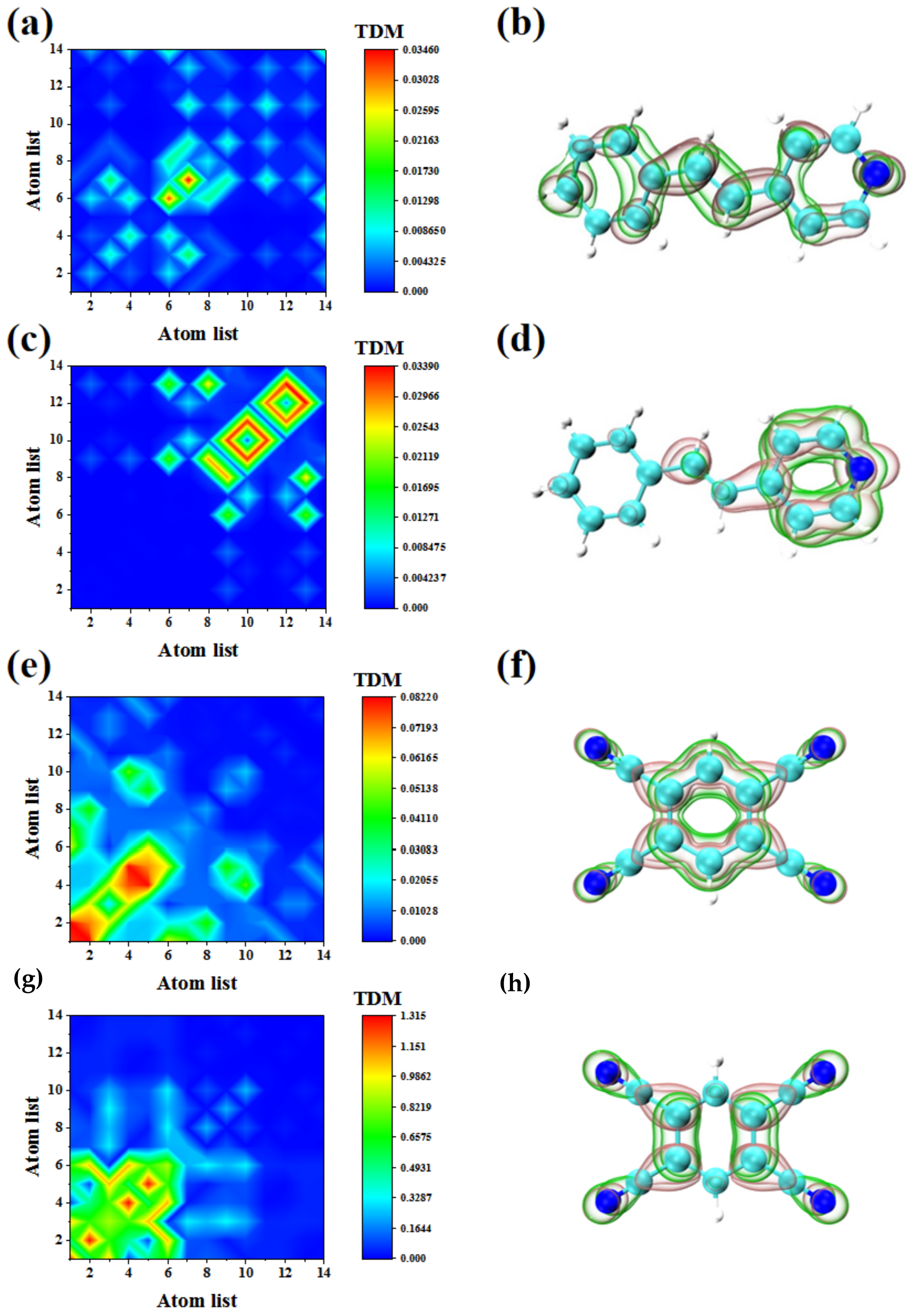

Figure S3. The monomer Spe's TDM and electron-hole pairs density of S_1 (a, b) and S_4 (c, d); the monomer TCNB's TDM and electron-hole pairs density of S_1 (e, f) and S_2 (g, h).
